# Machine Learning Model to Predict Assignment of Therapy Homework in Behavioral Treatments: Algorithm Development and Validation

**DOI:** 10.2196/45156

**Published:** 2023-05-15

**Authors:** Gal Peretz, C Barr Taylor, Josef I Ruzek, Samuel Jefroykin, Shiri Sadeh-Sharvit

**Affiliations:** 1 Eleos Health Waltham, MA United States; 2 Center for m2Health Palo Alto University Palo Alto, CA United States; 3 Department of Psychiatry Stanford Medical Center Stanford, CA United States

**Keywords:** deep learning, empirically-based practice, natural language processing, behavioral treatment, machine learning, homework, treatment fidelity, artificial intelligence, intervention, therapy, mental health, mHealth

## Abstract

**Background:**

Therapeutic homework is a core element of cognitive and behavioral interventions, and greater homework compliance predicts improved treatment outcomes. To date, research in this area has relied mostly on therapists’ and clients’ self-reports or studies carried out in academic settings, and there is little knowledge on how homework is used as a treatment intervention in routine clinical care.

**Objective:**

This study tested whether a machine learning (ML) model using natural language processing could identify homework assignments in behavioral health sessions. By leveraging this technology, we sought to develop a more objective and accurate method for detecting the presence of homework in therapy sessions.

**Methods:**

We analyzed 34,497 audio-recorded treatment sessions provided in 8 behavioral health care programs via an artificial intelligence (AI) platform designed for therapy provided by Eleos Health. Therapist and client utterances were captured and analyzed via the AI platform. Experts reviewed the homework assigned in 100 sessions to create classifications. Next, we sampled 4000 sessions and labeled therapist-client microdialogues that suggested homework to train an unsupervised sentence embedding model. This model was trained on 2.83 million therapist-client microdialogues.

**Results:**

An analysis of 100 random sessions found that homework was assigned in 61% (n=61) of sessions, and in 34% (n=21) of these cases, more than one homework assignment was provided. Homework addressed practicing skills (n=34, 37%), taking action (n=26, 28.5%), journaling (n=17, 19%), and learning new skills (n=14, 15%). Our classifier reached a 72% *F*_1_-score, outperforming state-of-the-art ML models. The therapists reviewing the microdialogues agreed in 90% (n=90) of cases on whether or not homework was assigned.

**Conclusions:**

The findings of this study demonstrate the potential of ML and natural language processing to improve the detection of therapeutic homework assignments in behavioral health sessions. Our findings highlight the importance of accurately capturing homework in real-world settings and the potential for AI to support therapists in providing evidence-based care and increasing fidelity with science-backed interventions. By identifying areas where AI can facilitate homework assignments and tracking, such as reminding therapists to prescribe homework and reducing the charting associated with homework, we can ultimately improve the overall quality of behavioral health care. Additionally, our approach can be extended to investigate the impact of homework assignments on therapeutic outcomes, providing insights into the effectiveness of specific types of homework.

## Introduction

Assigning homework (or “action plans”) for clients to complete between sessions is a key component of cognitive behavioral therapy and many other types of therapy. Behavior plans that encourage the between-session practice of therapy-relevant skills are a core element of time-limited interventions and are predictive of treatment outcomes [1-3]. Greater compliance with assigned homework predicts improved outcomes across a range of conditions, including anxiety, depression, substance use, posttraumatic stress disorders, eating disorders, obsessive-compulsive disorders, irritable bowel syndrome, and more [4-9]. Clients who had consistently completed homework were found to benefit significantly more from the intervention than those who had completed little or no homework [10]. However, little is known about whether or not therapists assign homework in customary practice settings.

Digital tools are changing the landscape of behavioral treatment delivery, training, and supervision. Recent technological developments in machine learning (ML) and artificial intelligence (AI) can help in the study of how evidence-based therapies are practiced in the field [11]. ML and AI are emerging technologies that are increasingly used to evaluate large data sets of mental health interventions. ML refers to algorithms and statistical models that can learn and adapt without explicit instructions by analyzing and drawing conclusions from data patterns [12]. Deep learning (DL) is a subset of ML in which computers learn to think using structures modeled on the human brain. AI are computer systems that capitalize on ML advancements and can perform tasks that typically require human intelligence, such as speech recognition, interpretation, and decision-making [13]. ML and AI have the potential to automatically detect trends in therapeutic exchanges and flag issues of interest that will expand the opportunity to assess and optimize service delivery in ways that can increase therapist fidelity and adherence to behavioral treatments and consequently improve treatment outcomes. These algorithms have great potential to inform and improve clinical work.

Until now, information on how therapists practice was limited to either data from controlled studies carried out in academic settings (which did not necessarily generalize to other treatment settings) or therapist self-reports [14]. While the evidence suggests that assigning therapeutic homework facilitates better outcomes in controlled studies, it is unclear how often this technique is used in real-world treatment settings.

Previous studies have relied on self-reporting by therapists and clients; however, self-reporting is subject to bias [15]. If natural language processing and ML systems can be trained to accurately identify homework assignments in behavioral health sessions as they are carried out in the field, it would enable a measurement approach with several advantages, including reducing the burden on therapists to report on homework completion, enabling real-time monitoring and tracking of homework assignments, and providing a more accurate and comprehensive understanding of the use of homework assignments in routine clinical care. Further, the novelty of our approach lies in our use of unsupervised sentence embeddings, which allow us to automatically identify and categorize homework assignments without the need for explicit labeling or supervision [16]. To this end, we endeavored to create an ML model that could accurately identify whether homework was assigned in a therapy session. Furthermore, we compared this model to other generic models that have not been uniquely used in the therapy domain.

## Methods

### Settings and Interventions

This study analyzed completely anonymized data from 34,619 audio-recorded outpatient treatment sessions provided in 8 behavioral health care programs across the United States. Clients sought treatment for a variety of mental health concerns, and therapists were allowed to use any treatment modality they believed was best for the client’s particular presenting problems. The data set used in this research includes 6236 clients and 675 therapists.

### The Eleos Health Platform

The Eleos Health platform was used to capture and process the session data. This is a digital platform that provides intervention feedback and supports clinical decision-making [17]. The platform captures essential indicators from treatment sessions and combines them with standardized assessment scales based on insights gained from treatment data sets analyzed using ML and natural language processing. The information is made available to therapists and supervisors [14,18,19].

### Ethics Approval

This study was approved by an independent institutional review board (Sterling Institutional Review Board), with expertise in behavioral health research (9545). All clients and therapists consented to share their deidentified data with the platform. All data storage was compliant with Health Insurance Portability and Accountability Act (HIPAA) guidelines, and only deidentified, aggregated data were used in this study.

### Preliminary Iterative Classification of Homework Assignment

To classify the types of conversations that are carried out in treatment and may suggest homework assignments, 4 therapists with graduate training in clinical psychology or clinical social work reviewed 100 treatment sessions randomly selected from the Eleos Health data set. Homework was defined as any task assigned to clients by their therapist to complete outside of the therapy session to enhance the therapeutic process. These domain experts determined whether (1) the session included a homework assignment or not and (2) which type of homework was assigned. Following the approach outlined by Hannah and Lautsch [20], the experts provided the initial codes independently and then convened at multiple points to compare, discuss, and reassess their coding. Once a consensus was reached, homework categories were coded. Further, 19 sessions (19%) were coded by more than one expert independently to assess the interrater agreement on whether the therapist had assigned homework or not.

### Data Set Organization

**Figure 1:**
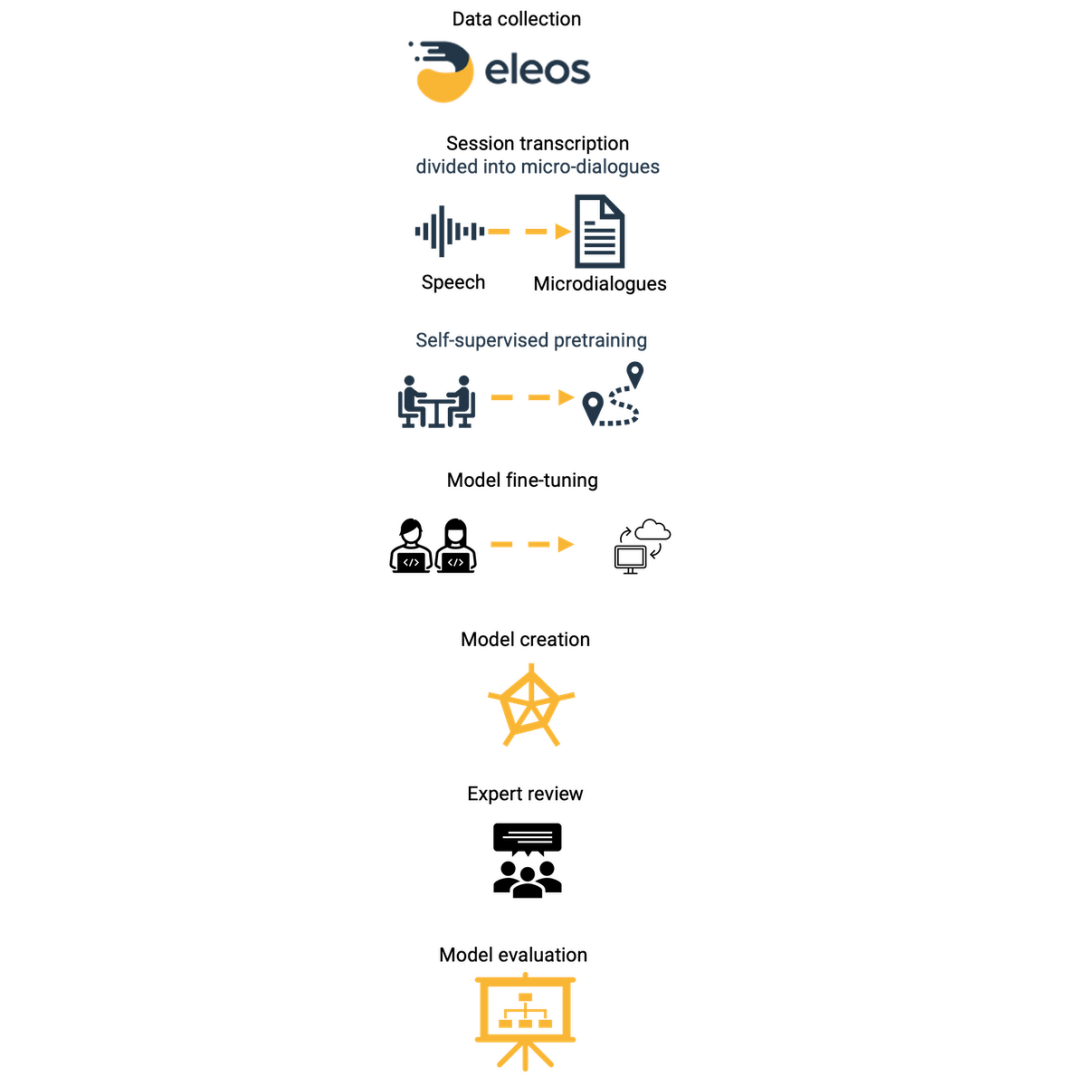
An overview of the data analysis process.

Figure 1 provides an overview of the data organization and analysis processes. Sessions were recorded and automatically transcribed, and the sentences were separated to differentiate therapist and client utterances using a proprietary automatic speech recognition and speaker diarization algorithm (Sadeh-Sharvit et al [14] describe the method). We first created a data set containing 4000 sessions that included homework-related search words and created for each a microdialogue segment, which was a short snippet of the therapist-client conversation, including a few sentences before and after the homework-related words. The 4 abovementioned domain experts rated these 4000 microdialogue candidates, indicating whether the conversation snippet included homework assignments or not and, if so, which type of homework was assigned. To train the model, we then split the data into training, validation, and testing subsamples (n=2800, 70%; n=400, 10%; and n=800, 20% of the 4000 snippets, respectively).

### Analytic Approach

**Figure 2:**
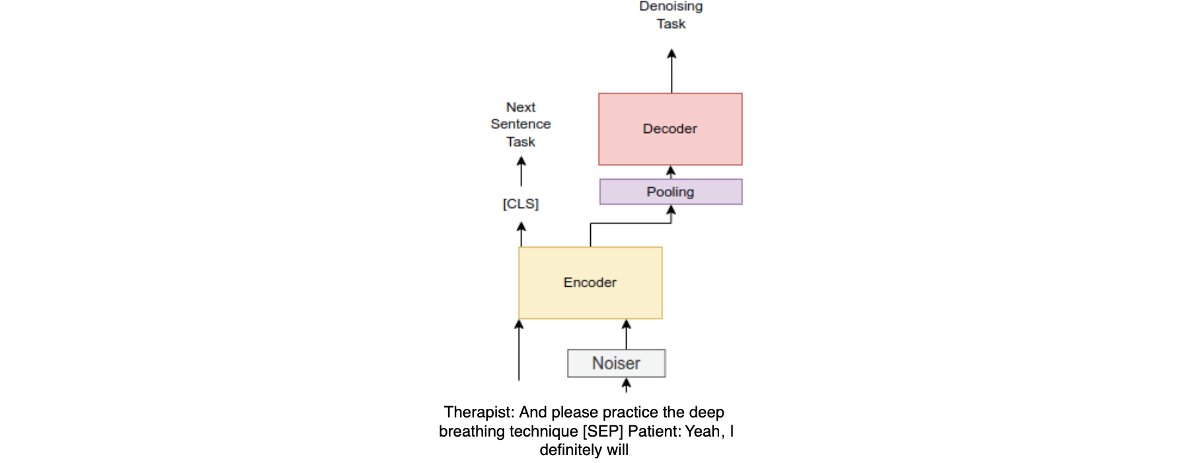
A summary of the self-supervision pretraining process on the treatment homework assignment. CLS: sentence-level classification; SEP: separator.

**Figure 3:**
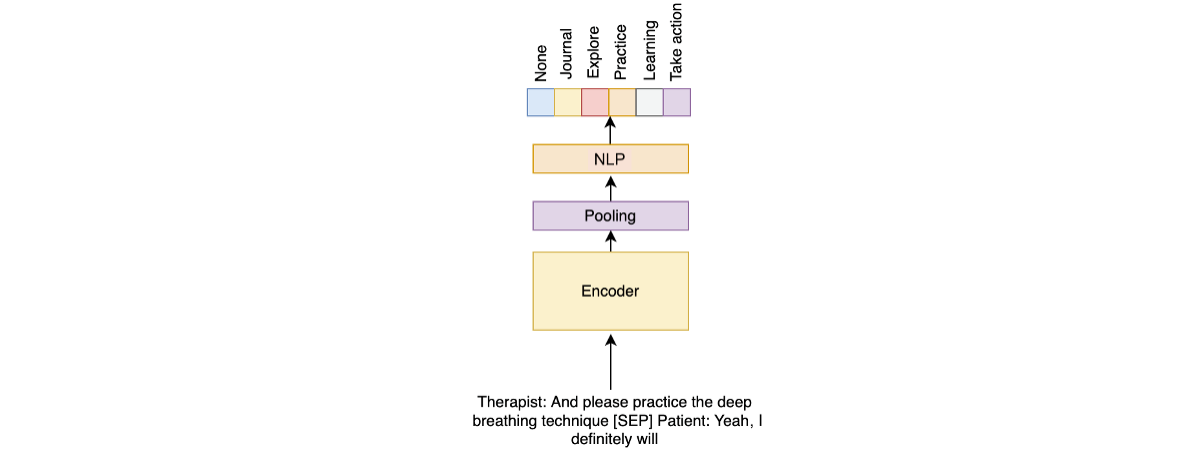
Model fine-tuning using a new set of parameters. MLP: multilayer perceptron; NLP: natural language processing; SEP: separator.

We used all the remaining treatment sessions in the data set (ie, 30,497 sessions) for testing the ML model. We initially parsed the sessions to generate 2.83 million microdialogues, in which we grouped any 2 sequential sentences said by therapists and clients to provide additional context to the model. Then, to create our final DL model, we split the training phase into 2 parts. The first part leveraged all the unlabeled conversations to train an encoder model that encoded the sentences to a good feature representation. We used the 2.83 million conversations to train the encoder model to grasp a good feature representation of the conversations between the therapist and client in a self-supervised manner. We started with the pretrained Bidirectional Encoder Representations from Transformers (BERT) model [21]. This was followed by adding a pooling layer to get a vector that represented features from the dialogue. We then added a decoder layer and trained an encoder-decoder model using a denoising technique (a summary of the approach is described by Wang et al [22]). In addition, we added a “next sentence prediction” task to a multitask learning setup. This helped the encoder learn a good representation of the dialogue and the dependency between the sentences of the different speakers. Figure 2 describes the pretraining process in detail. This model was trained using a V100 Tesla GPU (Nvidia Corp) over 3 days on 2.83 million microdialogues (ie, sequential sentences). After the self-supervision training process, we fine-tuned the encoder using the training part of the 4000 homework-related dialogues. We added 2 fully connected layers with rectified linear units (ReLUs) and dropouts to the encoder (Figure 3). We froze the weights of the encoder and then trained the model with a cross-entropy loss using the data set.

To further evaluate the Eleos homework model, we compared it to a wide range of state-of-the-art classification models. As a baseline model, we used the term frequency-inverse document frequency (TF-IDF) [23] and Doc2Vec [24] as feature extractors and combined them with a simple logistic regression model to evaluate the difficulty of the problem and understand if we needed a DL model to solve it. Additionally, we compared our model to the Robustly Optimized BERT Pretraining Approach (RoBERTa) [25] and Transformers and Sequential Denoising Auto-Encoder (TSDAE) [20] models without the next sentence loss to understand the benefit of adding this loss. To compare the models, we used an *F*_1_-score metric. This approach allowed us to balance the model’s precision and recall [26]. An *F*_1_-score score provides a robust and reliable evaluation metric for a model’s performance, particularly given the anticipated imbalanced nature of the data, with many sessions not including homework assignments. The *F*_1_-score was calculated by using the following formula:



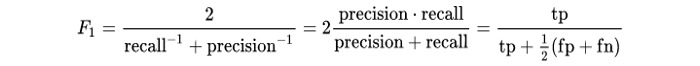



Finally, the results of the *F*_1_-score metric for each homework category were compared between Eleos’ homework model and the TF-IDF, Doc2Vec, RoBERTa, and TSDAE methods.

## Results

### Homework Classification

The initial iterative process that included the audio recordings of 100 randomly selected therapy sessions found that 61% (n=61) included at least one homework assignment. A total of 91 homework assignments were assigned, with the following categories emerging from the data.

#### Practicing (n=34, 37%)

These were sessions when therapists and the client planned that the latter would practice a technique that they were familiar with (eg, diaphragmatic muscle relaxation and dialectical behavioral therapy skills). For example, one therapist said, “It’ll help start calming your mind a little and setting yourself up for the day.... I’m thinking if you can try to do it at night.”

#### Taking Action (n=26, 28.5%)

In these sessions, the therapist and the client reviewed actions that the client could take outside the session to improve their mental health (eg, speaking with a partner about a sensitive topic, reaching out to their parole officer, or “unfollowing” social media accounts that are triggering nonadaptive behaviors).

#### Journaling (n=17, 19%)

In these instances, the therapist asked clients to express their emotions and describe their experiences through writing between the sessions. Very few therapists also mentioned self-monitoring of symptoms, and self-monitoring was therefore included as a form of journaling. Examples include the following: “I want you to journal your emotions. Write down your feelings connected to your grief” and “so your homework is going to be every time, I want you to write down every time you have a self-defeating thought.”

#### Learning (n=14, 15%)

In these sessions, the therapist recommended that the client use time outside the session to learn a new skill (eg, watch a YouTube video on self-compassion or complete a chapter in a self-help book). A therapist, for instance, made the following statement: “I’m gonna email you a copy of this. Okay. And you can start looking over it and we can talk about it.”

In 34% (n=21) of the sessions that included homework, more than one homework task was given. Of these instances, 81% (n=17) involved homework as a practicing assignment and another homework plan. The independent raters agreed 90% (n=90) of the time on whether or not homework was assigned. Raters tended to disagree mostly on whether discussions of skill practice outside the session were indeed homework assignments.

### Strength of the Eleos Homework Algorithm Classification

Table 1 outlines the results of the comparison using the *F*_1_-score for each model. The *F*_1_ metric found that the proposed model outperformed all the other ML models, especially in the “practice” and “learning” categories. Our classifier reached a 72% *F*_1_-score, on average, for all the categories and outperformed the baseline metrics for each category (Table 1).

**Table 1:** Results of the *F*_1_-score metric for each homework category.

Model	Practice	Take action	Learn	Journal	No homework	Overall score
TF-IDF^a^	0.6146	0.3124	0.4864	0.4329	0.5668	0.48262
Doc2Vec	0.6236	0.3973	0.5102	0.4926	0.5672	0.51818
RoBERTa^b^	0.6977	0.5112	0.6121	0.7129	0.7359	0.65396
TSDAE^c^	0.7132	0.5793	0.6630	0.7722	0.8236	0.71026
Eleos homework model	0.7407	0.5802	0.6712	0.7715	0.8312	0.71896

^a^TD-IDF: term frequency-inverse document frequency.

^b^RoBERTa: Robustly Optimized BERT Pretraining Approach.

^c^TSDAE: Transformers and Sequential Denoising Auto-Encoder.

## Discussion

### Principal Findings

Homework assignments and reviews are effective means of increasing treatment impact and extending the influence of psychological interventions beyond the treatment session conversation itself [27,28]. However, little is currently known about the extent to which psychotherapy delivered under naturalistic conditions includes the assignment of any homework and, if assigned, what the nature of the homework is. This study demonstrates the feasibility of determining rates and topics of homework assignments in routine care using a digital platform and creating an ML model to automatically identify homework discussions. To the best of our knowledge, this study is the first to develop and evaluate an ML model for identifying homework assignments in behavioral health sessions.

This study, which used 2.83 million therapist-client microdialogues, allowed for the development of an ML model that can predict therapeutic homework assignments with high accuracy based on natural language conversations between therapists and clients in real-world settings. We found that 61% of the sessions included at least one homework assignment. In light of the importance of homework for therapy outcomes, this number is reassuring and corresponds with previous studies [29]. The 34,619 sessions analyzed in this study were delivered by 675 therapists who likely followed a variety of theoretical models, as is often the case in real-world settings [30]. Some types of therapies (eg, nondirective or supportive therapies) would not be expected to include homework. The homework categories that emerged included practicing skills that had been reviewed in the session, taking constructive actions consistent with therapeutic goals, journaling and self-monitoring, and learning new information about the nature of one’s problems and strategies to address them. Of note, we found very few mentions of self-monitoring; therapists, rather, were more likely to provide a more general homework prompt to write down one’s feelings, experiences, and cravings. Future studies could explore therapists’ cognition and training regarding the greater use of journaling prompts compared to self-monitoring.

The results also suggest that the accuracy of our ML model showed high agreement with the classifications made by the expert raters and overperformed established ML models that had not been trained on large treatment data sets. These insights, derived from unobtrusive, passively collected therapy data, can be used to suggest more nuanced and empirically supported interventions [31]. Given how busy therapists are with multiple demands, including documenting, we wanted to find a technique that could easily provide feedback to therapists and treatment programs to the extent they wanted to consider this a best practice [32,33].

While there is data on treatment homework in controlled studies, this study suggests that it is also possible to determine if therapists assign homework in actual practice. We found that a therapy-specific ML model outperformed other models that had not been pretrained on data from treatments carried out in real-world settings. As more researchers and companies develop AI platforms, it is important to remember that ML models should be adjusted and trained according to the content matter they aim to capture. Given the less-than-optimal assignment of homework, the next question is how AI can improve this. AI can be used to help therapists determine if homework is required, to remind therapists to prescribe homework as part of best practices if needed, to help reduce charting associated with homework, and to nudge the therapists and clients about homework that needs to be practiced and reviewed. Furthermore, the method can also determine the impact of assigning homework at all, or the type of homework, on therapeutic outcomes, as these data are collected on the Eleos platform.

### Limitations

The limitations of this study include the absence of extended demographic data on the clients (eg, age, sex, race, or ethnicity), the reasons for therapy, and the treatment approach. These data do not permit an assessment of the nature, frequency, or quality of homework assignments as observed here in terms of best practices. In the future, the relationship between rates and types of homework assignments and treatment outcomes could be determined to inform recommendations about the use of this technique. From a methodological perspective, this study was not designed to isolate, observe, or diagnose erroneous ML predictions; however, we recommend that future studies include our technique as an additional analysis of the model’s performance. Future research should also leverage electronic health record data to refine the ML model. Studies could also assess whether a homework assignment that had been discussed was further reviewed in the following therapy appointment, whether homework was completed, and whether treatment review and homework completion are associated with improved clinical outcomes [32]. Therapist adherence to treatment protocols can be more difficult to achieve in routine care settings than in research studies, especially given the limited opportunities to observe therapist behavior as well as the increased demand for therapy and the accompanying increase in administrative tasks. As ML and AI continue to advance, there is great potential for them to be used to support therapists in assigning and tracking homework, ultimately leading to improved therapeutic outcomes. ML and AI can help increase therapist fidelity to empirically supported treatments in the field by enabling therapists (and supervisors) to more routinely self-monitor their use of key aspects of treatments (eg, homework assignments) during the therapy process. By leveraging the power of AI, therapists can be reminded to prescribe homework as part of best practices, reduce the charting associated with homework, and improve the overall quality of care they provide. The same techniques could also be used to examine other strategies (eg, cognitive restructuring) that are part of evidence-based treatments. Moving forward, it will be important for researchers and companies to continue developing and refining these AI platforms to ensure that they are tailored to the specific content matter they aim to capture, thereby paving the way for further advancements in this important field.

## Abbreviations

AI: artificial intelligence
BERT: Bidirectional Encoder Representations from Transformers
DL: deep learning
HIPAA: Health Insurance Portability and Accountability Act
ML: machine learning
ReLUs: rectified linear units
RoBERTa: Robustly Optimized BERT Pretraining Approach
TF-IDF: term frequency-inverse document frequency
TSDAE: Transformers and Sequential Denoising Auto-Encoder

## Data Availability

The data sets generated during and/or analyzed during this study are not publicly available due to privacy and compliance restrictions.
